# Program coordinators’ perspectives on implementing a transition program for newly graduated nurses: a qualitative interview study

**DOI:** 10.1108/JHOM-01-2023-0018

**Published:** 2024-04-01

**Authors:** Annika Eklund, Maria Skyvell Nilsson

**Affiliations:** Department of Social and Behavioural Studies, University West, Trollhättan, Sweden; Department of Health Sciences, University West, Trollhättan, Sweden

**Keywords:** Implementation, Healthcare organization, Newly graduated nurse, Transition program

## Abstract

**Purpose:**

While transition programs are widely used to facilitate newly graduated nurses transition to healthcare settings, knowledge about preconditions for implementing such programs in the hospital context is scarce. The purpose of this study was to explore program coordinators’ perspectives on implementing a transition program for newly graduated nurses.

**Design/methodology/approach:**

An explorative qualitative study using individual interviews. Total of 11 program coordinators at five acute care hospital administrations in a south-west region in Sweden. Data was subjected to thematic analysis, using NVivo software to promote coding.

**Findings:**

The following two themes were identified from the analysis: Create a shared responsibility for introducing newly graduated nurses, and establish legitimacy of the program. The implementation process was found to be a matter of both educational content and anchoring work in the hospital organization. To clarify the what and why of implementing a transition program, where the nurses learning processes are prioritized, was foundational prerequisites for successful implementation.

**Originality/value:**

This paper illustrates that implementing transition programs in contemporary hospital care context is a valuable but complex process that involves conflicting priorities. A program that is well integrated in the organization, in which responsibilities between different levels and roles in the hospital organization, aims and expectations on the program are clarified, is important to achieve the intentions of effective transition to practice. Joint actions need to be taken by healthcare policymakers, hospitals and ward managers, and educational institutions to support the implementation of transition programs as a long-term strategy for nurses entering hospital care.

## Background

Although far from a new phenomenon, transitioning from the student role to engaging effectively in the nursing role is often an exciting yet challenging process (Dyess and Sherman, 2009; Murray *et al*., 2019). Newly graduated nurses (NGN) struggle to adjust to a new professional self, responsibilities and routines and to become a member of the working group (Andersson *et al*., 2022; Duchscher, 2009), while at the same time being expected to perform at higher levels despite being a novice practitioner (Missen *et al*., 2014). The transition experiences are suggested to influence the retention of NGNs in the profession and workforce. Newly graduate nurse turnover is costly and destabilizing for healthcare organizations (Phillips *et al.*, 2015; Rush *et al*., 2019). A Swedish study found that one in five nurses strongly intended to leave the profession after five years in the profession and the levels of those intentions increased during the first years of employment (Rudman *et al*., 2014). Many studies report NGNs indicate that their intention to remain within the nursing profession is related to their satisfaction with transition (see, e.g. Detlín *et al*., 2022; Phillips *et al*., 2014). Addressing these issues and creating supportive learning and working conditions calls for strategic long-term actions. As an example, transition programs have been implemented in hospital care settings. Although studies have repeatedly highlighted the value and necessity of such programs from the perspective of NGNs, our knowledge of establishing, implementing and developing these programs from an organizational perspective is scarce.

Although workers learn through participation at work, learning through everyday practice alone may not be sufficient to maintain currency of knowledge and sustain employability (Billett and Choy, 2013). Research has suggested that nursing leadership can increase nurses’ integration in work and intention to stay by meeting their needs for competence development and organizing for career development (Karlsson *et al*., 2019; Baumann *et al*., 2018). In addition, formal or informal strategies to help new employees learn the knowledge, skills and actions they need to succeed in the organization they just entered (Phillips *et al.*, 2015). Such activities could include reduced workload, support material to grasp routines or more comprehensive interventions such as transition programs.

### Overview of the transition program literature and aim

With the aim of providing a safe and supportive transition for NGNs, transition programs have been implemented in different care settings around the world over the past few decades (see, e.g. Hampton *et al*., 2021; Wildermuth *et al*., 2020). These programs were designed to bridge a perceived gap between student experiences and working life as a registered nurse, and more recently as a vehicle to attract and retain NGNs. Such programs are defined as formal agreements between the NGN and the employer, outlining and defining responsibilities, and attention to new nurses as they advance into the profession (Alsalamah *et al*., 2022; Eklund *et al*., 2021). These programs are typically constructed around various educational components, such as structured ward introduction, including supervision, reflection seminars, lectures, simulations, clinical rotations and mentorship, to capture a variety of the transition experience. Review studies have reported benefits for both the NGN and their employing organization as, in addition to providing clinical experience, such programs can strengthen self-confidence, job satisfaction and communicative skills and reduce stress (Rush *et al*., 2019; Hampton *et al*., 2021). Structured programs that combined elements of building competence and confidence, with elements of educational strategies (i.e. simulation), socialization and embedding the novice nurse into the work environment, using preceptorship or mentoring may also be most effective at retaining nurses (Brook *et al*., 2019) and positively influence the NGNs transition experience (Rush *et al*., 2019). Such programs have also been reported to have lasting effects on workforce integration and retention (Baumann *et al*., 2018; Letourneau and Fater, 2015). These aspects contribute to the motives of implementing transition programs for hospitals searching for ways to manage nursing staff shortages and enhance the retention of new nurses.

There is an extensive and growing body of literature that explores the elements and effectiveness of transition programs to support learning and retention. Still, several reviews reported the issue of variability in the focus, definitions and evaluation measures of the programs, that in turn can impact how well the programs support the needs of the NGNs as well as the implementation (Bakon *et al*., 2018; Rush *et al*., 2019; Kenny *et al*., 2021). In addition, a review by Aldosari *et al.* (2021) reported that the efficacy of these programs and their impact on NGNs’ transition experiences, competence and retention is unclear, since few studies employ validated measures or use control groups. Improvements cannot necessarily be credited to transition program participation alone, as other studies suggested that NGNs gradually improve with time and experience (Duchscher, 2009). Furthermore, implementing interventions or innovations in health care is expected to contribute to change, but has been found to be a challenge in complex healthcare organizations (see, e.g. Jacobs *et al*., 2015). Much of the research on transition programs has explored the abovementioned aspects from the perspectives of the NGNs, whereas there is limited knowledge on other stakeholders’ perspectives. A study by Missen *et al*. (2014) pointed out transition program coordinators’ as having a crucial role in providing appropriately planned strategies to support NGNs. The aim of this study is to contribute to this knowledge field by exploring program coordinators’ perspectives on implementing a transition program in hospital care in a Swedish region. Their perspective on and experience of working with the program can be a valuable contribution to understanding how hospital organizations can organize support strategies for NGNs during early professional life.

## Methods

### Setting

The study was conducted in a south-west region in Sweden, with approximately 3,800 hospital beds, with five publicly funded hospital administrations. One is a university hospital, and the others are emergency hospitals and county hospitals. The program has two overall process leaders, and each administration has one program coordinator. These also form a regional working group, with approximately eight meetings per year, to share experiences from the transition programs at their administrations. The program coordinators have smaller teams within their administration working with specific lecture content, educational components or administrative tasks, although this arrangement varies slightly across the administrations.

The 12-month transition program was developed to support a safe and secure transition for all NGNs, irrespective of care specialty at their wards, with less than four months of work experience as nurses after graduating from a three-year bachelor’s program, and to secure the required nursing competence and retention (Eklund *et al*., 2021). All NGNs were permanently employed and all activities in the program were conducted during paid working hours. The program was successively introduced at the hospitals since 2015, has been mandatory from the regional level since 2018. The program is regionally funded, such as program administration and training sessions, and formally administered from each separate hospital administration. At four of the hospital administrations, the program is administered from the hospital’s education units, while one is administered from the Human Resource (HR) unit.

A regional decision document provides general guidelines for the hospital administrations about the overall structure and components. The program structure and components are like many of those described in the international literature (Bakon *et al*., 2018; Rush *et al*., 2019), including introduction and supervision at the ward, education days (lectures, practical work and/or simulations), reflection seminars in group with other NGNs, clinical rotation or auscultation, and mentorship. However, it is not stipulated how each component or educational content should be delivered. The content and number of lectures (6–12) varied across hospital administrations; hospitals using simulations arranged fewer lectures. The number of days or periods for clinical rotation at other wards at the hospital also varied and were related to the individual NGNs interests and possibilities for rotation in the organizations. The specific knowledge, skills and routines required at the wards are expected to be trained at the ward where the NGN is employed.

### Data collection and analysis

Eleven key informants involved in implementing and managing the transition program, which was a total sampling of those employed in the region as responsible for implementing the program. These participants were program coordinators (seven), regional process leader (one) and education managers or responsible for the educational content (three) were selected from two of the hospitals due to their organization of the program where they work closely to the program coordinators regarding the program activities, with a clear mandate and involvement in the program. In one case, the program coordinator was also the education manager. All participants were employed at the hospital administrations’ HR or education units, and the program coordinators were responsible for the implementation of the program at the respective hospitals. The participants had no overall management responsibility; although they were responsible for executing the transition program, they were unable to oversee the work performed to introduce the NGNs at the wards. The regional process leader was the convener of a regional group where the program coordinators met approximately eight times a year, to follow up and develop the program jointly. Three of the program coordinators had recently left their position at the time for the interview, but were still partly involved in the transition program and included due to extensive experience. All the participants were also registered nurses. For readability, all participants will be referred to as program coordinators in this study. The authors had previously evaluated the regional transition program and its components from a learning perspective, where contact with the coordinators was established. Hence, for this study, the authors had no dependent position to the participants, and the participants were not involved in the design, purpose, interpretations or conclusions.

All interviews were individual, except one that was conducted in pairs since they wanted to because they shared the same function at the hospital. The authors conducted five interviews each. The interviews were conducted online using Zoom, and lasted between 30 and 84 min (average 55 min). The interview guide contained questions such as “Describe the process when the program was first introduced at your hospital”, “Describe your role and responsibilities in the program”, “How well-established is the program at your hospital?” and “How do you understand your mandate regarding the content of the program?”. The interviews were recorded and transcribed verbatim. The data was thematically analyzed (Fereday and Muir-Cochrane, 2006) using NVivo software to promote encoding. First, the authors read through the transcripts separately to gain a deeper understanding of the content. Second, the authors jointly identified passages that reflected the coordinators’ understandings of the program implementation and development were selected and sorted into nodes. The nodes were sorted into categories that were built and refined throughout the analysis. During this process, the authors returned to the original data to double-check the context of the utterances. Specific attention was given to categories and nodes where disagreement or uncertainty arose between the authors regarding how the categories reflected a specific node. This process resulted in four categories. Subsequently, the authors discussed meaningful clusters of the information, from the aspect of what the categories included and excluded, as suggested by Castleberry and Nolen (2018) to clarify the coherence. This process resulted in the final two themes, each representing “an organizing concept—a shared core idea” (Clarke and Braun, 2017, p. 297), to inform the study’s findings of participants’ perspectives on implementing a transition program. Both authors were involved in the whole analysis process. The COREQ criteria for reporting qualitative studies were used (Tong *et al.*, 2007).

### Ethical considerations

The study was ethically approved by the Swedish Ethical Review Authority (DNR: 2021–02788). The participants gave their written informed consent after being informed orally and in writing how the data would be handled and presented, and that their participation was voluntary, with the option withdraw at any time without giving a reason. The transcriptions were provided with codes and kept separate from the audio file.

## Results

The analysis revealed two themes illustrating the implementation process of a transition program in a hospital context, from the perspectives of program-coordinators (see Table 1).

### Create a shared responsibility for introducing newly graduated nurses

This theme captures the participants description of implementing the transition program as a way of bridging the gap between undergraduate education and hospital work. This was covered by the category *Identifying and managing learning needs*, reflecting that much of the coordinator’s implementation work was to identify and arrange activities to meet the NGNs’ learning needs and expectations from the wards. Implementing the program was further described in the category *Broaden the view of introduction*, where the coordinators strived to emphasize the NGNs’ learning and a more comprehensive role developing processes in a context where introduction traditionally mainly focused ward-specific routines and clinical work at the separate wards. The implementation of was, however, also clearly impacted by preconditions and workload at the wards.

#### Identifying and managing learning needs

This category reflects the participants’ work with identifying and responding to NGNs’ learning needs. The most prominent motive for implementing the program was that the NGNs often lacked the readiness and specific skills needed for hospital work. Consequently, participants described that one of their main responsibilities of implementing the program was to ensure appropriate content of lectures and seminars. The learning content was, to a large extent chosen by the coordinators, based on either what the NGNs needed advanced training in or knowledge gaps from undergraduate education related to the specific hospital routines. The content was successively modified from the input from the regional group of coordinators, NGNs and ward managers. An example was that after a few years, lectures on recovery was added as an activity to support the NGNs well-being.

The participants expressed an expectation from nursing ward managers that the program should be implemented to support specific skills development needed for daily work. This originated from a widespread perception among ward colleagues and managers that undergraduate education cannot fully prepare the NGN for practical work in a highly specialized and effective hospital care. The participants requested closer cooperation with the educational institutions to ensure appropriate content and activities for the program to be implemented as a bridging function between education and early working life. However, the expectations from the wards were for the program to be implemented to fill a range of specific knowledge gaps from undergraduate education:

There was an expectation that the transition program would solve everything. We received inquiries about whether we could teach the nurses to insert a peripheral venous catheter … but they must know that before they get to the program and if they don't, they have to learn it on the ward (X3)

The participants further understood that the strength of the program was largely based on the variety of program components, where meeting and exchanging experiences with peers and more established employees in group supervision, specific lectures and skills training were emphasized. Implementing a variation of learning activities was understood to broaden the introduction to the nursing role and create security:

You have supervisors who are available, you have mentors, and you have process-oriented nursing supervision. Then you provide security on different levels, both in the practical care work but … also in your role, but also your socialization. (X4)

How the participants managed the task of identifying learning needs reflected their general views on learning, which also mirrored their dedication to understand what it is like to be a novice nurse, understand their context and their individual needs, and to encourage the NGNs to step forward and use their knowledge. To organize activities for developing the relational aspects of work were emphasized as essential to facilitate the NGNs transition:

I believe that you find security in interpersonal communication, when meeting other people, the meeting in the work group, with relatives, with patients. That increases security more than security of how to put an intravenous drip or manage certain medical equipment (X6)

#### Broaden the view of introduction

The implementation of a transition program on a regional basis was described as an ongoing process to broaden how the NGNs were introduced in their profession. Traditionally, the wards were responsible for the introduction and typically focused on ward-specific routines and their specific patient groups. Implementing the program added learning opportunities for the NGNS to meet, share experiences and practice with other NGNs across different nursing specialties. Still, some of the activities are conducted at the wards (e.g. introduction to ward-specific routines and supervision), while others were conducted outside the wards (e.g. simulations). This could create ambiguity regarding division of responsibilities between the wards and program coordinators for implementing a sufficient introduction, but also that the wards to some extent lose control of the introduction:

The interesting question is for the wards, not at the management level but for the ward level. They want to have more power and control over the introduction themselves, and that it was taken away from them. You have to let employees go to participate in an introduction that you cannot control, and you cannot niche it so that it fits your own ward. (X6)

At the same time, the program coordinators had no impact on the ward-specific introduction, even though it was counted as a part of the transition program. Here, the participants emphasized the importance of the program being implemented as a strategy to respond to the NGNs needs of introduction and learning, rather than being implemented as a separate activity that is primarily used as a way of solving the turnover issue. Still, another related dimension was the challenges for the wards to create a balance between learning and managing daily work due to the staffing situations:

There were some wards where they chose not to leave or made it difficult for the nurses to leave for the program activities. It was a goal conflict that was directly problematic for the new employee (X3)

The goal conflict also required the participants to be responsive regarding the activities in the program. For example, clinical rotation was initially implemented to provide a broader introduction to hospital care. This component has been removed or modified over the years at most of the hospitals with the motive that it caused insecurity in many NGNs to start over with new routines, patient groups and colleagues and that the ward lost a trained nurse. Hence, such adjustments were also said to hamper the path to the program as providing a changed view of introduction, in terms of short-term planning focusing on daily staffing rather than a long-term strategy building a broader competence for NGNs.

### Establishing legitimacy of the program

This theme illustrates how the implementation of a transition program requires legitimacy across different levels at the hospitals and region. The category *A coordinating function with mandate* reflected the importance of having specific function at each hospital administration that could oversee and be the voice for the implementation and development of the program. A lack of anchoring across different levels in the hospital organization meant that the implementation was vulnerable and dependent on the coordinators’ mandate, knowledge and motivation to progress. The category *A joint commitment* reflected the participants understanding that all levels at the hospitals must pull in the same direction to support the NGNs learning processes and role development.

#### A coordinating function with mandate

The participants emphasized the importance of the coordinator, or the group working with the program at each hospital, having a mandate to make decisions about the program content. The motives were the different pre-requisites at the hospitals, such as the numbers of NGNs, access to simulation training and where the program responsibility was located. To take on a coordination function, the participants further described the importance of continuously putting the program on the agenda:

The biggest advantage is that there is someone who runs it all the time (…) and to constantly make sure that it is included when there is reorganization. ‘Hey, we need to think about the transition program and how it is affected’. There's always someone … I'm like a lawyer for the program. (X4)

Further, the participants framed their function as being responsive to expectations and needs from a range of actors: the central region, hospital management, ward managers and NGNs. They described a great variety of tasks in their function, including presenting and discussing the program for hospital boards and ward managers, arranging supervisors for process-oriented seminars, deciding and developing the educational content, and carrying out evaluations of the activities. In addition, the program coordinator could be a neutral part for the NGNs to share challenges from their ward with and to support them to prioritize the program activities:

Many may not themselves see the value of stopping, ‘you just have to work, work, work’. It’s not easy when you are new to stand up for yourself and see what is unreasonable (X1)

The participants often returned to the individual-dependency and personal engagement for how the program was organized and the success of implementation. The regional steering group with all program coordinators was often emphasized as important for the cohesion of the program. The participants specifically valued this group as an opportunity to share experiences about the implementation process and how they arranged the educational activities and to discuss possible changes in the program.

However, the organization around the program was also understood to be vulnerable since both coordinators and ward managers had been replaced over the years. The knowledge and engagement were understood as largely being carried by individuals, which was an obstacle for both implementation and continuous development of the program. The participants addressed the need to anchor the program across different functions and levels at the hospitals:

It has probably been like that to get it rolling; you have enthusiasts to work with it, but you can't just build on that. Everyone must be able to carry the program in the organization where it is managed (X5)

#### A joint commitment

The initiative for the regional program came from a steering group in the central region. Those participants who had been involved in developing the initial concept for the program described that the process “went at a furious pace” (*X10*). However, the coordinators expressed the importance of the program being funded and implemented as mandatory by the central region. The obligation was used as a basis for arguing for a prioritization on learning and safe transition for the NGNs. However, they experienced a general limited knowledge about and priority for the program in the hospital management, or guidance on how to implement it at the hospitals. This was described to cause a gap between the regional governance that introduced the program, and the coordinators and ward managers who were set to effectuate it. The gap led to the program being not perceived as a joint effort across managing levels in the hospitals. As an example, generally, few or no adjustments were made in the organizations; for example, the nurses are fully scheduled as ordinary staff with little or no dedicated time for the program activities:

Our organization does not support the transition program, it has just been added to the existing organization and existing structure (X8)

An important aspect of the implementation was for the coordinators to continuously provide the ward managers with knowledge about the program, to understand the aim and value of the program for the NGNs and their wards.

Although the transition program is mandatory for the hospitals to organize and for the NGNs to join, there was an awareness that not everyone follows this path. Some NGNs are employed at a ward without being assigned to the program, some are prevented from joining, and some do not appear in the activities. This means the opportunities for learning when entering the profession vary. In addition, the coordinators reported that it was relatively easy to cut out the program activities if daily work at the ward needed to be prioritized. This was particularly evident during the COVID-19 pandemic, but had also been an issue previously. To change this situation, the coordinators described a need for clear descriptions of the execution of the program and anchoring in the hospital management. To clarify the program as a joint commitment, they further argued for a more clearly stated and coordinated structure of the program to clarify the intentions and expectations of the program for hospital management, ward managers and NGNs:

I would like a more coordinated system … that the framework is set (…) Maybe not that we have the same content, which lecturers to be involved and so on, but that in some way we still talk about transition program in the region, well then “this is what you get” (X7a)

The participants compared the program with the general medical training program (for newly qualified physicians), where learning activities are more well-established, and strategies for learning were perceived to not be discussed as contradictive with everyday work. Therefore, a regional joint governing framework was emphasized to solve the logistic issue, so the nurses get to leave the ward work to engage in the program activities. However, it should be noted that a complete standardized program was understood to complicate and slow down processes of changes in the program if needed. The request for regional governance was a question of making the program a matter of course with clearly set goals, rather than details regarding the content:

The important thing is that the goals are clear, maybe not how we get there in the first place (X8)

## Discussion

This study aimed to explore the implementation of a transition program for NGNs from the perspective of program coordinators. Implementing a program was understood as acknowledgement of the NGNs’ need for structured support and learning when entering working life. The process turned out to be a continuous matter of content, structure and anchoring work. In line with Painter *et al*. (2019), the results reflect that not only is the implementation dependent on a well-designed program or curricula considerations, but must also be understood against the background of the hospitals’ organization and preconditions. The programs are implemented and evaluated in a context where limited organizational and workload adjustments are made to create space for learning. Here, in line with Billett and Choy (2013), we argue that a broader understanding of the workplace learning environment will assist those responsible for organizing transition programs in facilitating learning while meeting the changing performance requirements. According to the study’s findings, we advocate for the design of transition programs that draw from an understanding of the novice experience during the transition phases, to effectively bridge the undergraduate curricula with workplace expectations to successfully integrate NGNs (Duchscher, 2009). Facilitating transition experiences involves providing novices with roles and responsibilities commensurate with their knowledge and confidence levels, ensuring consistent workplace support, and fostering familiarity and success in meeting expectations related to care delivery and skill performance (Duchscher and Windey, 2018). Against this background, the implementation could benefit from clarifying what parts of the introduction that could be arranged for within the program and what parts should be arranged at the wards. This could reduce the present ambivalence of the program being an education activity set out to develop specific skills or as a broader transition program (Phillips *et al*., 2015).

Most of the hospitals organized the transition program from their education units, while one was organized from their HR unit. This revealed ambiguity across different levels in the hospital organizations regarding the goals of implementing the program: as an introduction to learning specific hospital ward skills and to the professional role as a hospital nurse, but also for employment and retention. Thus, clarification of the overall aim of the program; *what* the program should comprise and *how* the different components contribute to the overall aim of the program, and *why* (the motives for implementation) could clarify the expectations on the program, and the responsibilities for various parts of NGNs transition. These issues relate to the fact that there are many actors and levels with different interests and motives involved in the implementation, such as hospital management, ward managers, employees at the wards, HR, the educational institutions, the NGNs and the coordinators. As pointed out by Sarkies *et al*. (2021), the interdependence of these levels within the healthcare system determines which strategies are more likely to successfully implement the change that the program involves. The tension between different motives illustrated in the results indicates that the actors could benefit from developing knowledge created at the boundaries of practices (Edwards, 2020). Put differently, they share the goal that the program should support both learning and retention that no actor will solve alone. Still, the actors struggle with partly competing priorities of managing daily clinical work and longer-term aspects of developing as a professional and retention, which has been highlighted as challenging implementation in health care (Jacobs *et al*., 2015). The conflict of goals arises in the meeting between what the stakeholders are facing to solve right now and tools for developing professional roles in the long term (Eklund *et al*., 2021). In addition, COVID-19 was a stressor that has shed extra light on this goal conflict and the vulnerability of implementing training efforts. Here, implementation strategies for learning not only require the individual actors’ responsiveness and intense work, but, as suggested by Alsiö *et al*. (2022), adequate structural and organizational cultural conditions that include all levels in the hospital system, including the educational institutions.

A transition program is accompanied by a fundamental shift in individual and organizational consciousness and values (Kramer *et al*., 2012; Jacobs *et al*., 2015). It entails a change of the prevailing vision of the reality of nurses’ work in hospitals, including a shift from tasks to what and why is achieved in practice. The ambivalence between implementing a fully structured, general program and degrees of freedom could be troublesome, since the evidence highlights the value of structured and uniform (Rush *et al*., 2019; Hampton *et al*., 2021), and theoretically grounded programs (Graf *et al*., 2020). Still, other research suggests that hospitals should develop internal programs since preconditions and individual needs vary (Cline *et al*., 2017). Here, a program coordinator can be understood as an important function for creating and maintaining collaborations across stakeholders in the organizations to keep together the structure and content. They are agentic and their efforts have a clear impact on how the program was implemented and developed, yet they often found themselves struggling due to the organizational demands (Edwards, 2020). Hence, this study illustrates the difficulties of implementing a structured and mandatory program from the regional level, but where local adjustments are often made due to workload (meaning, e.g. activities could be reduced or that the NGNs drop out, but without any consequences). Such a goal-conflict points to an emphasis on organizational priorities and joint commitment to support the NGNs during transition, where any adjustments should be motivated by specific needs for learning and availability of facilities. The request for regional governance to support the implementation could be understood as the program coordinators requiring support to manage the goal conflict between supporting the NGNs transition process and adjusting to the wards demands to manage daily care.

### Limitations

The results of this study should be interpreted with certain limitations in mind. The participants included are from the same regional organization, representing the same program framework, which means that other ways of structuring and organizing the program might have different implications for implementation. Another limitation, and as highlighted by others as a general issue in the transition program research (Kenny *et al*., 2021), is that the program content varies to some extent, which might impact transferability. However, the research design, setting, methods for data collection and interpretation are clearly described, and quotations are included in the results to enhance the possibility to judge its credibility and potential transferability.

The interviews were conducted online using Zoom, which might to some extent reflect the variation of length of the interviews. Online procedures may have influenced that the sharing of experiences and nuances might have been lost (Oliffe *et al.*, 2021). Still, the longer interviews were with those who had worked with transition programs for a long time, meaning the length might rather reflect their vast experiences.

## Conclusion

The journey from being a student to a professional novice nurse in a hospital context can be inspiring, challenging and require abilities to learn, while at the same time managing daily care; aspects that seems to also be true for implementing a transition program. Such competing priorities deserve further attention. Findings from this study offer insights into the complexities of implementing transition programs that is under constant pressure to remain in a form that supports the NGNs transition, and not the day-to-day requirements of health organization staffing and nursing practice. The goal conflict between short- and long-term responsibilities and ambitions in a hospital organization are fundamental challenges for the implementation of transition programs. This suggests that attention needs to shift to developing the organization in which the programs are effectuated. A transition program that is integrated in the organization, where responsibilities across management levels and roles in the organizations are clear and agreed upon, and where aims and expectations on the program are clarified, could support the implementation. Thus, the involved actors could progress in their ambitions toward a structural change to support the NGNS while transitioning to and developing in their profession.

### Implications for practice

Given an increasing interest in transition to practice and the significant financial investment in multifaceted transition programs, the result of this study presents valuable knowledge for nursing management about challenges and preconditions for implementation. Actions and prioritizations need to be taken by healthcare policymakers, hospital and ward managers and educational institutions to support the implementation of transition programs as a long-term strategy to support the transition of newly graduated nurses. Choosing to either better integrate the transition program in the ward work *or* focusing the program on activities outside the wards, could clarify both the intentions and responsibilities of the program. Further, building a team of different competences and functions in the hospital organizations could be a way to create a sustainable program and reduce dependency of each individual coordinator, ward manager and NGN to carry the transition.

## Figures and Tables

**Table 1:** Overview of the themes, categories and nodes

Theme	Create a shared responsibility for introducing newly graduated nurses		Establish legitimacy of the program	
Categories	Identify and manage learning needs	Broaden the view of introduction	A coordinating function with mandate	A joint commitment
Nodes	Connection to higher education institutions Curricular framework Handle knowledge and skill gaps Handle knowledge expectations Providing a broader professional perspective	Introduction to the profession vs ward Prioritize learning vs everyday care Adjusting to workload	Put the program on the agenda A function between the ward and NGN Vulnerable organization Personal engagement	Regional governance Mandatory Anchoring on all levels A framework with degrees of freedom for local adjustments
